# A Collision Relationship-Based Driving Behavior Decision-Making Method for an Intelligent Land Vehicle at a Disorderly Intersection via DRQN

**DOI:** 10.3390/s22020636

**Published:** 2022-01-14

**Authors:** Lingli Yu, Shuxin Huo, Keyi Li, Yadong Wei

**Affiliations:** 1 School of Automation, Central South University, Changsha 410083, China; 194611075@csu.edu.cn (S.H.); li_keyi88@sina.com (K.L.); 13477011934@163.com (Y.W.); 2 Hunan Xiangjiang Artificial Intelligence Academy, Changsha 410000, China

**Keywords:** deep recurrent Q network, intelligent land vehicle, decision-making, collision relationship, partially observable Markov decision process

## Abstract

An intelligent land vehicle utilizes onboard sensors to acquire observed states at a disorderly intersection. However, partial observation of the environment occurs due to sensor noise. This causes decision failure easily. A collision relationship-based driving behavior decision-making method via deep recurrent Q network (CR-DRQN) is proposed for intelligent land vehicles. First, the collision relationship between the intelligent land vehicle and surrounding vehicles is designed as the input. The collision relationship is extracted from the observed states with the sensor noise. This avoids a CR-DRQN dimension explosion and speeds up the network training. Then, DRQN is utilized to attenuate the impact of the input noise and achieve driving behavior decision-making. Finally, some comparative experiments are conducted to verify the effectiveness of the proposed method. CR-DRQN maintains a high decision success rate at a disorderly intersection with partially observable states. In addition, the proposed method is outstanding in the aspects of safety, the ability of collision risk prediction, and comfort.

## 1. Introduction

An intelligent land vehicle makes driving behavior decisions based on environmental information through sensors. However, sensor noise is inevitable owing to natural factors such as weather [1], temperature, or road conditions. For example, the detected positions of surrounding vehicles are deviated by road inclination. Moreover, there is noise in the data obtained by sensors because of the vehicle’s movement and the sensors’ structure [2]. Thus, the data acquired by sensors may increase, decrease, or even be lost. This difference between observed states and environmental states causes a wrong estimation of environmental conditions. For instance, a surrounding vehicle is very close to the ego vehicle, while the detected distance data is still within the range of the safe distance. In this case, it is easy to make wrong decisions based on inaccurate states of surrounding vehicles. Furthermore, this may lead to traffic accidents, traffic congestion, and inefficiency [3].

The driving behavior decision-making is responsible for selecting an appropriate driving behavior according to a planned path and the environmental states [4]. The driving behavior refers to the target position, or the target speed, or the target acceleration, which is one decision period ahead of the current state of the intelligent land vehicle on the path. Then, the result of driving behavior decision-making is sent to the trajectory planning part. Usually, the performance of the driving behavior decision-making is evaluated in terms of safety and comfort [5]. However, the environment in the real world is full of uncertainty. As a result, the environmental states are always partially observable [6]. Therefore, this study focuses on the driving behavior decision-making problem with partially observable states.

At present, the driving behavior decision-making method attracts wide attention. This mainly includes the game theory method [7,8], generative decision method [9], fuzzy decision method [10,11], etc. A game theory-based decision-making model for lane changing in urban congested intersections is presented in [7]. The model considers the cooperation between the intelligent vehicle and adjacent vehicles before a lane change. In addition, taking the conflict between safety, efficiency, and comfort into account, an intelligent vehicle decision-making model based on game theory is proposed to select the optimal driving strategy [8]. These methods model the decision-making process as a game by simplifying the environment and ignoring the uncertain factors in the environment. In the aspect of generative decision-making, the finite state mechanism is used for a high-accurate parking detection to eliminate the interferences from adjacent vehicles [9]. Although the method is very interpretable, it is very difficult to generate a complete rule in a complex environment. In terms of fuzzy decision making, Cueva et al. [10] designed a fuzzy behavior decision-making method to improve the efficiency of the vehicle sensor information exchange. Moreover, Balal et al. [11] designed a lane change decision system based on binary fuzzy reasoning for the highway environment. However, the accuracy of the membership degree directly determines the accuracy of the decision estimation inescapably, and the design of membership functions still depends on the human experience.

The partially observable Markov decision process (POMDP) is a suitable model for the environmental states under sensor noise. POMDP is utilized to estimate the behavior of other traffic participants and gives a safe trajectory to the self-driving vehicle [12,13]. Silva et al. [12] presented a data-driven machine-learning method for classifying driving styles and provided automated feedback to drivers on their driving behaviors. Moreover, a data-driven method is proposed to predict vehicles’ short-term lateral motions for safety decision-making [13]. These methods have excellent search and analysis capabilities for the environmental states, so they can better deal with the complex and uncertain environment. Furthermore, deep reinforcement learning (DRL) based on POMDP is an effective method for decision-making [14,15], because it studies naturalistic driving data or driving expert experiences to achieve more human-like driving behaviors. Li et al. [14] built a mapping relationship between the traffic image and the vehicle operations and obtained an optimal driving strategy of the vehicle based on the deep Q network (DQN) at the intersection. DQN also yields robust performance in lane and speed change decisions while an intelligent vehicle gains noisy observation [15]. To conclude, the common decision-making method is summarized in Table 1.

In addition, the recurrent neural network (RNN) is gradually applied in the domain of intelligent land vehicles. Sallab et al. [16] applied a recurrent neural network combined with an attention mechanism for information integration, to process partially observable driving scenes. The long short-term memory (LSTM) based on RNN is utilized to predict the future state of the surrounding vehicles for motion planning [17]. A deep recurrent Q network (DRQN), a combination with DQN and LSTM, is also adopted to solve the problem of traffic light control [18,19]. LSTM is a group of networks with loops in them and retains memory about the previous state [20]. This can train time series and reduce the influence of the noisy input. As a result, a combination of DRL and LSTM can be well applied to driving behavior decision-making for intelligent lane vehicles in a noisy environment.

In this study, a collision relationship-based driving behavior decision-making method for intelligent land vehicles based on DRQN (CR-DRQN) is put forward. This method solves the problem of instability in decision-making caused by decreased perceptual confidence successfully. The main contributions in this paper are:

- A collision relationship-based driving behavior decision-making method for intelligent land vehicles is put forward. The collision relationship between an intelligent land vehicle and surrounding vehicles is utilized as the state input, rather than the positions and velocities of all the vehicles. This effectively avoids dimension explosion of the network’s input with the increase in surrounding vehicles. Therefore, this design helps to make right decisions quickly.
- By using long short-term memory (LSTM) to train the time-series input, the proposed method effectively weakens the adverse effects of reduced perception confidence. Further, this method ensures the safety of driving behavior decision-making.
- A series of comparative simulations are carried out for a scene of disorderly intersection. The experiments verify that the proposed algorithm is superior to traditional DQN and its variants in the safety and comfort of decision-making.

The rest of this paper is organized as follows. First, related work is briefly reviewed in Section 2. Then, Section 3 introduces the foundation of deep reinforcement learning. Section 4 elaborates on the proposed method and the specific design for the observed states, action space, and reward. The simulation configuration and comparative results are shown in Section 5. Finally, the conclusion and future work are presented in Section 6.

## 2. Related Work

An intersection, especially one with no signal lights, is a typical uncertain and complex environment. It is a great challenge for intelligent land vehicles to make appropriate driving behavior decisions in this environment. Aimed at environmental uncertainty, Iberraken et al. [21] proposed a flexible and safe autonomous decision-making system, which improves the efficiency and security of decision-making for intelligent land vehicles. For complex traffic at intersections, Noh [22] proposed a probabilistic collision threat assessment algorithm, and Li et al. [23] established a dynamic safety potential field to describe the spatial distribution of vehicle-driving risks affected by the environmental state. In addition, Galceran et al. [24] proposed a synthesis reasoning and decision-making method in autopilot mode. CNN detection and Kalman filtering are used to predict the movement intention of obstacles as the basis for human-like, decision-making strategies [25]. This enhances the interaction between intelligent land vehicles and other drivers.

Two typical frameworks of DRL are based on policy gradients and value function. The deep deterministic policy gradient algorithm (DDPG) is a policy gradient-based deep reinforcement learning method suitable for continuous action space [26]. Huang et al. [27] used DDPG to map vehicles’ driving states, such as velocity and road distance, to driving behaviors, such as steering, acceleration, and braking. Moreover, Chen et al. [28] combined positive and negative rewards with the priority experience replay method. This effectively improves the sampling efficiency and enhances the performance of the DDPG model. To consider passenger comfort while ensuring safety, a multi-objective reward function is designed to study autonomous braking decision-making strategies based on DDPG in emergencies [29]. In addition, given the inconsistency between behavioral decision-making and trajectory planning, the dual-delay deep deterministic strategy gradient algorithm (TD3) is used to solve the optimal decision strategy, and the route feature is extracted from the path planning space as the behavioral decision-making state space [30].

DQN is a deep reinforcement learning method based on value function, which can effectively solve discrete action space problems [31]. Kai et al. [32] used the DQN algorithm to obtain an optimal driving strategy considering safety and efficiency. Further, Chen et al. [33] combined DQN and fuzzy algorithm to deal with the correlation between different motion commands. This makes the network results more feasible. In addition, Kamran et al. [34] designed a risk assessment strategy as a reward for DQN, rather than a judgment about whether a collision occurs or not. In addition, DQN’s variants are also applied in the field of driving behavior decision-making. To reduce the impact of environmental uncertainty, a dual-channel attention module is designed to enhance the analysis ability of the environmental state. Then, the module is integrated into the dueling double deep Q network (D3QN) to make safer and more efficient decisions for autonomous driving [35]. Mokhtari et al. [36] utilized two long-term short-term memory (LSTM) models based on a double deep Q network (DDQN) and the priority experience replay method to reconstruct the perception state of the environment and the future trajectories of pedestrians.

## 3. Foundation of Deep Reinforcement Learning

In this section, POMDP under sensor noise is introduced first. This is the basic model of driving behavior decision-making for intelligent land vehicles. Then, an overview of deep reinforcement learning is provided.

### 3.1. Partially Observable Markov Decision Process under Sensor Noise

At a real intersection, sensor noise creates a difference between the environmental states and the observed states. However, POMDP is suitable for the agent in an uncertain scenario. Thus, to represent a partially observable environment, a driving behavior decision-making method for intelligent land vehicles is modeled by POMDP [37].

POMDP is expressed as a six-tuple 〈S,A,T,R,O,Ω〉 [38]. *S* is an environmental state set while *A* is an action set, including a series of driving behaviors. *T* is the state transfer function. *R* is the reward function. *O* is a set of observed states. Ω is the observation model. $o_{t}\text{\textasciitilde{}}O(s_{t})$ shows that an intelligent land vehicle receives observed states $o_{t}$ instead of environmental states *S_t_* in step *t*.

The observed states are detected by onboard sensors. These include an ego vehicle’s position $\left[x_{0},y_{0}\right]$, an ego vehicle’s velocity $v_{0}$, and surrounding vehicles’ positions $\left[x_{i},y_{i}\right]$. It is assumed that only the ego vehicle’s states are completely observable. This means that the ego vehicle’s observed position and velocity are the same as the real values. The surrounding vehicles’ observed positions are defined as Equation (1). It adds noise to the environmental positions with a certain probability, and the value of the noise is not fixed. This design is closer to the real environment.

(1) $$O([x_{i},y_{i}])=\{\begin{array}{c} {}[x_{i},y_{i}]+L_{\mathrm{err}}\times f_{gauss}(x)\times rand(-1,0,1),if\;c<\tau \\ {}[x_{i},y_{i}],else \end{array}\;$$

*c* is a random variable within [0, 1]. When the probability of noise occurrence τ is greater than *c*, some noise randomly plays a part in the surrounding vehicles’ observed positions. $L_{\mathrm{err}}$ represents the observation error. $f_{gauss}(x)$ is a gauss number.

### 3.2. Deep Reinforcement Learning

During reinforcement learning, the intelligent land vehicle learns a strategy π by interacting with the environment at the disorderly intersection to make driving behavior decisions. The state-action value function $Q_{\pi }(s,a)$ represents the performance of a given strategy π when choosing an action *a* in a state *s*. Thus, $Q_{\pi }(s,a)$ is denoted as:

(2) $$Q_{\pi }(s,a)=E_{\pi }\left[\sum_{k=0}^{\infty }\gamma^{k}R_{t+k+1}|S_{t}=s,A_{t}=a\right]$$

Q-learning algorithm maximizes the state-action value in Equation (2) to learn the optimal strategy π*. The optimal $Q_{\pi *}(s,a)$ follows the Bellman optimality equation:

(3) $$Q_{\pi *}(s,a)=E_{\pi *}[R_{t+1}+\gamma \underset{a\prime }{\max}Q_{\pi }(S_{t+1},a\prime )|S_{t}=s,A_{t}=a]$$

However, the Q-learning algorithm makes useless calculations when facing continuous and high-dimensional state input. As a powerful nonlinear function approximator, a deep neural network solves well the above problem.

The deep neural network is a perceptron model, trained by the backpropagation algorithm. The parameters of the network are adjusted by the gradient descent algorithm. In general, the loss function of deep reinforcement learning is defined as:

(4) $$L(\omega )=\frac{1}{2}{\left(R+\gamma \max_{a^{\prime }}Q(s^{\prime },a^{\prime };\omega^{-})-Q(s,a;\omega )\right)}^{2}$$

L(ω) is the variance between the target value and predicted value. $R+\gamma \max_{a^{\prime }}Q(s^{\prime },a^{\prime };\omega^{-})$ represents the target value, while Q(s,a;ω) represents the predicted value. An online Q network and a target Q network are constructed to calculate the predicted value and target value, respectively. To improve the stability of the algorithm, the target Q network’s parameters are updated with a fixed number of steps by copying the online Q network’s parameters. Besides, an experience replay memory is set up to store training samples. The online Q network is trained by randomly selecting samples from memory. This setup breaks the correlation of successive samples.

## 4. Collision Relationship-Based Driving Behavior Decision-Making via DRQN

In this section, the collision relationship between an intelligent land vehicle and surrounding vehicles is designed as the input of CR-DRQN. Then, CR-DRQN is utilized to determine the best strategy for driving behavior decision-making. The design of the decision-making model and the structure of CR-DRQN are described as follows.

### 4.1. Design of the Driving Behavior Decision-Making Model

To apply CR-DRQN to driving behavior decision-making at a disorderly intersection without a traffic light, the state space, action space, and reward function are designed as follows.

#### 4.1.1. State Space

The state space is defined as the collision risk between the ego-vehicle and surrounding vehicles: $\phi ={\left[\phi_{1},\phi_{2},...,\phi_{N}\right]}^{T}$. *N* is the number of surrounding vehicles. In this study, three surrounding vehicles from different directions have the probability of collision with the ego vehicle at a disorderly intersection, as an example.

**Figure 1:**
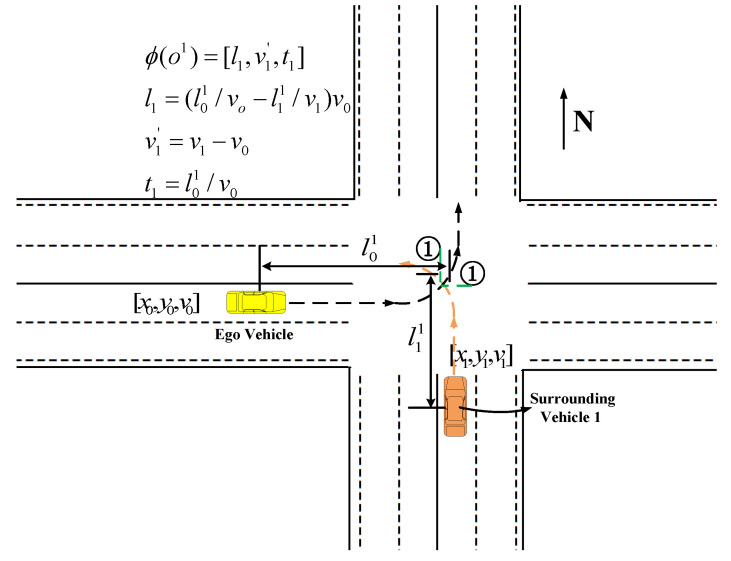
Description of collision relationship between an ego vehicle and one surrounding vehicle.

In Figure 1, the yellow car is the ego vehicle, while the orange car is the surrounding vehicle. The dashed lines from the ego vehicle and the surrounding vehicle represent driving paths. Further, the meeting point of two green dashed lines represents the vanishing point of the collision relationship. When the ego vehicle or surrounding vehicle leaves the intersection, the collision relationship disappears.

Take the collision relationship between the ego-vehicle and one surrounding vehicle as an example. When there is a collision relationship, the input state is defined as $\phi (o^{i})=[l_{i},v_{i}^{\prime },t_{i}]$. $o^{i}$ is the observed state of the surrounding vehicle *i*. $l_{i}$ is the safety distance between the ego vehicle and the surrounding vehicle *i*:

(5) $$l_{i}=(l_{0}^{i}/v_{0}-l_{i}^{i}/v_{i})v_{0}$$

where $l_{0}^{i}$ represents the arc length from the current position of the ego vehicle to the collision vanishing point *i*, and $l_{i}^{i}$ is the arc length between the surrounding vehicle *i* and the collision vanishing point *i*. $v_{i}^{\prime }$ is the velocity of the surrounding vehicle *i* relative to the ego vehicle. $t_{i}$ is the time that the ego vehicle uses to move from the current position to the collision vanishing point *i*: $t_{i}=l_{0}^{i}/v_{0}^{}$.

**Figure 2:**
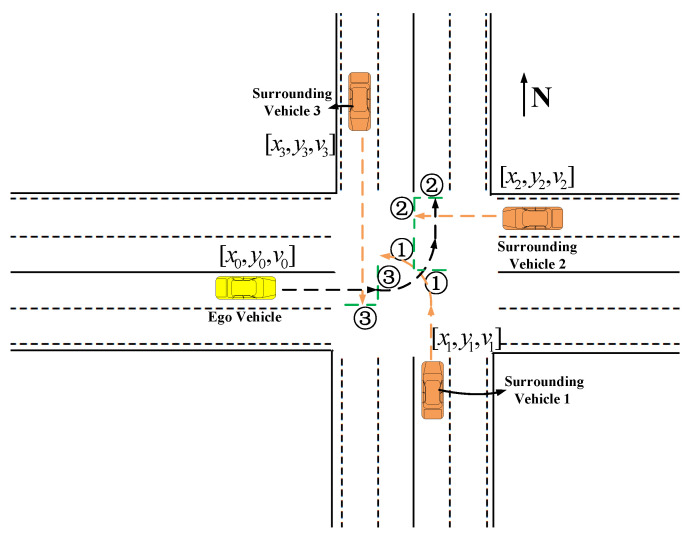
Description of collision relationship between an ego vehicle and multiple surrounding vehicles.

Similarly, the collision relationship between the ego vehicle and other surrounding vehicles is shown in Figure 2. In this case, the input state is a set of the collision relationship ϕ(o). Surrounding vehicles are counted counterclockwise in the stand of ego vehicle: the south surrounding vehicle is 1, the east surrounding vehicle is 2, and the north surrounding vehicle is 3. The dashed green lines ①, ②, ③ are the corresponding collision disappearance boundaries.

The advantages of this specific state setting are as follows. If the input state of the deep neural network is simply defined as the group of the position, course angle, and the velocity of the ego vehicle and surrounding vehicles, the quantity of state input is too huge. In the process of training and normalized calibration, it is hard to conduct enough training for each state. This may result in numerical problems. On the contrary, the application of the collision relationship contributes to simplifying and normalizing the network input. This setting of the input state not only avoids numerical problems but also improves the training speed and generalization ability of the network.

#### 4.1.2. Action Space

In this study, the ego vehicle only makes an acceleration decision without considering the temporary lane change behavior of surrounding vehicles. Action Space is expressed as:

(6) $$A{=[\mathrm{AS},\mathrm{AF},\mathrm{DS},\mathrm{BR},\mathrm{MA}]}^{T}$$

The specific meaning is as follows. AS refers to accelerate slowly while AF is accelerate fast. DS means decelerate slowly. BR refers to braking and MA represents maintenance. To ensure stability and comfort in driving, the action is maintained during every decision step.

#### 4.1.3. Reward Function

Three evaluation criteria determine the performance of CR-DRQN driving behavior decision-making. The reward function *R* in Equation (7) is defined by the mix of safety, comfort, and task completion efficiency:

(7) $$R=\alpha_{1}R_{safe}+\alpha_{2}R_{comfort}+\alpha_{3}R_{efficient}$$

α is the weight of each evaluation criterion.

*R_safe_* represents a safety reward and *L_safe_* represents the minimum safe distance. The definition of *R_safe_* is illustrated in Equation (8). On one hand, if *l_i_* is larger than or equal to *L_safe_*, the collision between the ego vehicle and the surrounding vehicle is unlikely to occur. In this case, *R_safe_* is set to be 1. On the other hand, if *l_i_* is smaller than *L_safe_*, there is the possibility of a collision. Thus, *R_safe_* is set as *−K*_1_ in this case, but no collision occurs. Furthermore, if the safety distance is short enough so that the collision happens, *R_safe_* is set as *−K*_2_. In addition, the relation between *K*_1_ and *K*_2_ is $K_{2}>K_{1}>0$. This is because a greater penalty is deserved due to the collision occurrence.

(8) $$R_{safe}=\{\begin{array}{c} 1,l_{i}\geq L_{safe}\\ -K_{2},collision\\ -K_{1},l_{i}<L_{safe}\underset{}{}\;\mathrm{and}\;\underset{}{}\mathrm{no}\;\underset{}{}collision \end{array}$$

*R_comfort_* refers to the comfort punishment shown in Equation (9). The velocity control expects a smooth process from acceleration to deceleration. If consecutive actions are acceleration and deceleration, the comfort punishment is negative.

(9) $$R_{comfort}=\{\begin{array}{c} -\Delta at,\mathrm{if}\;A=(\mathrm{AForAS})\text{\&}\mathrm{last}\;A=(\mathrm{BRorDS}),\;\mathrm{or}\;\mathrm{swap}\\ 0,else \end{array}$$

*R_efficient_* represents a task completion efficiency reward. It effectively prevents the ego vehicle from stopping at the stop line until there is no risk of collision in any case. Therefore, *R_efficient_* is designed as the velocity of the ego vehicle, which is presented in Equation (10):

(10) $$R_{efficient}=v_{0}$$

### 4.2. Driving Behavior Decision-Making Method Based on CR-DRQN

In POMDP, DQN fails to be a good approximation of state-action value function, because Q(o,a;ω)≠Q(s,a;ω). In this study, LSTM replaces the first full connection layer of DQN. LSTM is an improved recurrent neural network. The original RNN is very sensitive to short-term input because its hidden layer has only one state. However, the interval of the related input state under sensor noise is too long to be learned by the original RNN. This is called the long-term dependence problem. LSTM adds a cell to store the long-term state and expands the whole state according to the time dimension. It solves the long-term dependence problem that RNN cannot handle.

**Figure 3:**
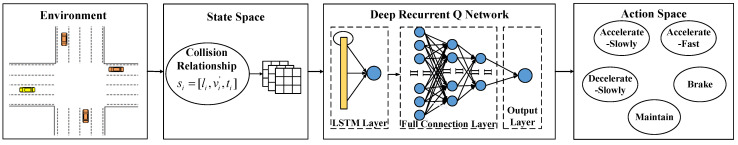
Construction of Driving Behavior Decision-making Method based on CR-DRQN.

The input state of CR-DRQN is the collision risk between the ego vehicle and surrounding vehicles. The construction of the CR-DRQN network is divided into three parts, as shown in Figure 3. The first part is the LSTM layer, the second is a full connection layer, and the last is the output layer. CR-DRQN outputs the state-action value of each action. The action with the maximal state-action value is selected at each step. Activation functions in the full connection layer are rectifier nonlinear activation functions (ReLU), while LSTM uses tanh and sigmoid functions, and the output layer uses the linear function. The pseudo-code of the CR-DRQN algorithm is presented in Algorithm 1.
**Algorithm 1:** CR-DRQN pseudocodeInitialize replay memory D with capacity NInitialize online Q network with parameters ω randomlyInitialize target Q network with parameters $\omega^{-}=\omega$For episode =1:M do Initialize observed state $o_{1}=O(s_{1})$ For t =1:T do  With probability ε select random action $a_{t}$, otherwise select $a_{t}{=\mathrm{argmax}}_{a}Q(\phi (o_{t}),a;\omega )$  Execute action $a_{t}$ in emulator and get reward $r_{t+1}$ and next observed state $o_{t+1}$  Store transition $\left(o_{t},a_{t},r_{t+1},o_{t+1}\right)$ in D  Set $y_{j}=\{\begin{array}{c} r_{j+1},\mathrm{if}\;\mathrm{episode}\;\mathrm{terminates}\;\mathrm{at}\;\mathrm{step}\;j+1\\ r_{j+1}+\gamma \max_{a^{\prime }}Q^{\prime }(\phi_{j+1},a^{\prime };\omega^{-}),otherwise \end{array}$  Update network parameters ω by using the gradient descent of ${\left(y_{j}-Q(\phi_{j},a_{j};\omega )\right)}^{2}$  Every C steps reset $Q^{\prime }=Q$ End forEnd for

## 5. Simulation Results and Discussions

In this section, experiments were conducted to verify the effectiveness of the proposed driving behavior decision-making method, compared with DQN [14], the combination of DQN and priority experience replay method (Prioritized-DQN) [28], DDQN [36], and D3QN [35]. Firstly, the environment and parameter settings are described. Next, the performance of the proposed method is revealed from the aspects of safety, the ability of collision risk prediction, and comfort.

### 5.1. Experiment Settings

In this section, the simulation environment is built to realize CR-DRQN and contrast algorithms, in Linux-based python by the Keras framework [39]. Meanwhile, their performances are compared at the disorderly intersection.

The initial velocity of the ego vehicle is 10 m/s (the velocity at the intersection is limited to 8.3 m/s), while the velocities of the surrounding vehicles are randomly selected from 10 m/s, 8 m/s, 6 m/s, 0. When the distance between the surrounding vehicle and the intersection is more than 150 m, the ego vehicle cannot detect a surrounding vehicle. 

Safety is the primary goal of vehicles driving at a disorderly intersection. Therefore, the average value of deceleration is slightly higher than that of acceleration, which is set as follows:

- If the ego vehicle takes the accelerate slowly action, acceleration *a* is +1 m/s^2^.
- If the ego vehicle takes the accelerate fast action, acceleration *a* is +3 m/s^2^.
- If the ego vehicle takes the decelerate slowly action, acceleration *a* chooses −2 m/s^2^.
- If the ego vehicle takes the brake action, acceleration *a* is set to −4 m/s^2^.
- If the ego vehicle takes the maintain action, acceleration *a* is 0.

Reward settings are as follows: *L_safe_* = 15, *K*_1_ = 5, *K*_2_ = 100. The decision period is *T* = 0.1 s and the weight of each evaluation criterion is $\alpha_{1}=\alpha_{2}=1,\alpha_{3}=0.2$.

**Figure 4:**
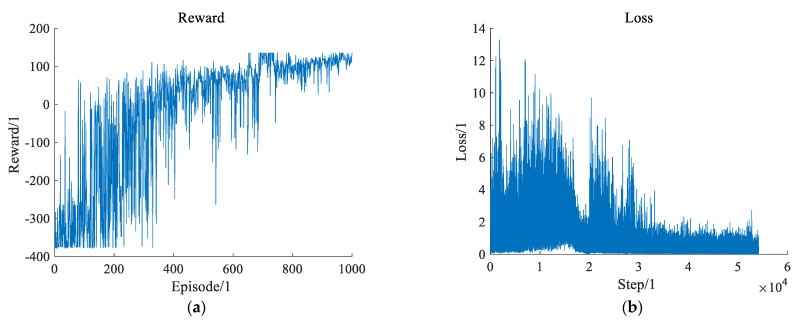
Training process based on CR-DRQN. (**a**) presents the change of the reward obtained by the intelligent vehicle with the increase of episodes. (**b**) presents the relationship between the loss and update steps.

Each training episode includes a series of decision steps with a period of 0.1 s. On one hand, Figure 4a shows a relationship between the cumulative reward and training episodes. The cumulative reward improves with the increase in training episodes and becomes stable in the end. On the other hand, Figure 4b shows a relationship between the network’s loss and update steps. The loss decreases gradually as the update steps develop. The loss comes to convergence too. To conclude, both indicate that the system has reached a stable state. That is, the ego vehicle learns to make decisions to avoid a collision with surrounding vehicles and drives through a disorderly intersection safely. 

### 5.2. Settings of CR-DRQN’s Network Layers and Neurons

The numbers of neural network layers and neurons are crucial factors for the network training [39]. These all affect the performance of CR-DRQN. If the parameters are too few, the neural network cannot come to convergence quickly. If there are too many parameters, the neural network appears to have an overfitting phenomenon easily. Therefore, 16 sets of commonly used network parameters are utilized to find a relatively better set of parameters.

According to the design of the existing work [40] and the references [28,41,42], the number of network layers is set to 4–8, considering the input dimensions of the network. Moreover, the number of neurons in each layer is generally designed to be a power of 2 or multiples of 10, and is halved layer by layer. The number of neurons in the last layer is the dimension of the action space.

Table 2 shows the performance of passing through an intersection for an intelligent lane vehicle under different network parameters. Q-value is the weighted sum of *R_safe_*, *R_comfort_*_,_ and *R_efficient_*. First, *R_safe_* stands for a safe distance from the point of collision. When the safe distance is greater than the minimum safe distance, it returns *R_safe_* = 1. However, *R_safe_* is negative as a punishment in the condition that the distance between the ego vehicle and the environmental vehicle is less than the minimum safe distance or the collision occurs. Then, *R_comfort_* guarantees a smooth driving velocity and provides a comfortable driving experience. Finally, *R_efficient_* assures that the ego vehicle can drive through the intersection. This also prevents the ego vehicle from slowing down to 0 in front of the intersection in any condition, until there are no surrounding vehicles. It is reasonable to set *R_efficient_* for an intelligent land vehicle.

The number of layers and neurons of each neural network is shown in Table 2. The network with the 9th group of network parameters completes the decision-making task and gains better performance than the others. 

This study uses bootstrapped random to update the weights of CR-DRQN. All the networks are trained by using the Adam algorithm with a learning rate of 0.001. The replay memory has a size of 2000 and the update interval of the target network is 100. The discount factor is set as 0.95 and the batch size for sampling is 32.

### 5.3. Performance of Comparative Experiments with Different Sensor Noise

A series of comparative experiments are conducted to illustrate the performance of CR-DRQN from three aspects: safety, the ability of collision risk prediction, and comfort. Among them, safety is first evaluated by the success rate of decision-making, because safety is the most crucial indicator of driving behavior decision-making. Then, to explore the reason for the high success rate, the ability of collision risk prediction is assessed. Finally, the comfort of CR-DRQN is verified. In addition, to present the perceptual confidence fluctuation, the experiments consider the probability of noise occurrence within 0–70%. In detail, sensor noise is set by varying the difference between the detected positions of surrounding vehicles and their actual values.

#### 5.3.1. Safety Evaluation

**Figure 5:**
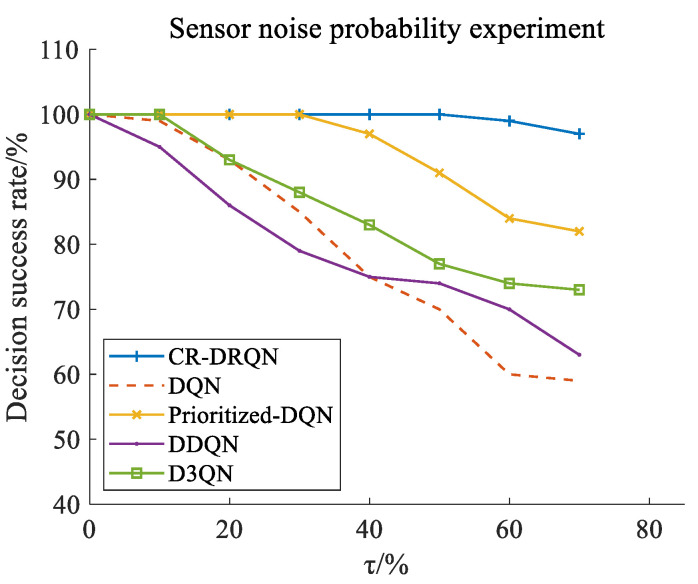
Decision success rate under different probabilities of noise occurrence.

In this study, the success rate is used to evaluate the safety of driving behavior decision-making. With different probabilities of noise occurrence, the model evaluation experiments are repeated 20 times, and each experiment contains 200 episodes. The experimental results are shown in Figure 5. The success rates of DQN, Prioritized-DQN, DDQN, D3QN, and CR-DRQN in decision-making decrease with the increase in probability of noise occurrence τ at a disorderly intersection. This illustrates that τ plays a role in the experiments. Additionally, the decline in the decision success rate of DQN exceeds that of DDQN when sensor noise probability is more than 40%. This shows that DQN is weaker than DDQN in dealing with the environment with many noises. Moreover, the result of D3QN is better than DQN and DDQN, but worse than CR-DRQN. Although D3QN combines the advantages of dueling DQN and DDQN, which can reduce the variance and solve the overestimation problem, it does not have an advantage in the state input with noise. Further, the success rates of CR-DRQN and Prioritized-DQN are almost the same, which are much higher than those of DQN, DDQN, D3QN with a low probability of noise occurrence. However, Prioritized-DQN’s success rate decreases obviously when the probability of noise occurrence is greater than 30%, while the success rate of CR-DRQN is still high. This is because LSTM trains the time-series input effectively and makes a critical effect under sensor noise. Furthermore, a high success rate needs a high ability of collision risk prediction, especially in the case of sensor noise occurrence. Therefore, the great performance of CR-DRQN in collision risk prediction is verified in the next subsubsection.

#### 5.3.2. The Ability of Collision Risk Prediction 

**Figure 6:**
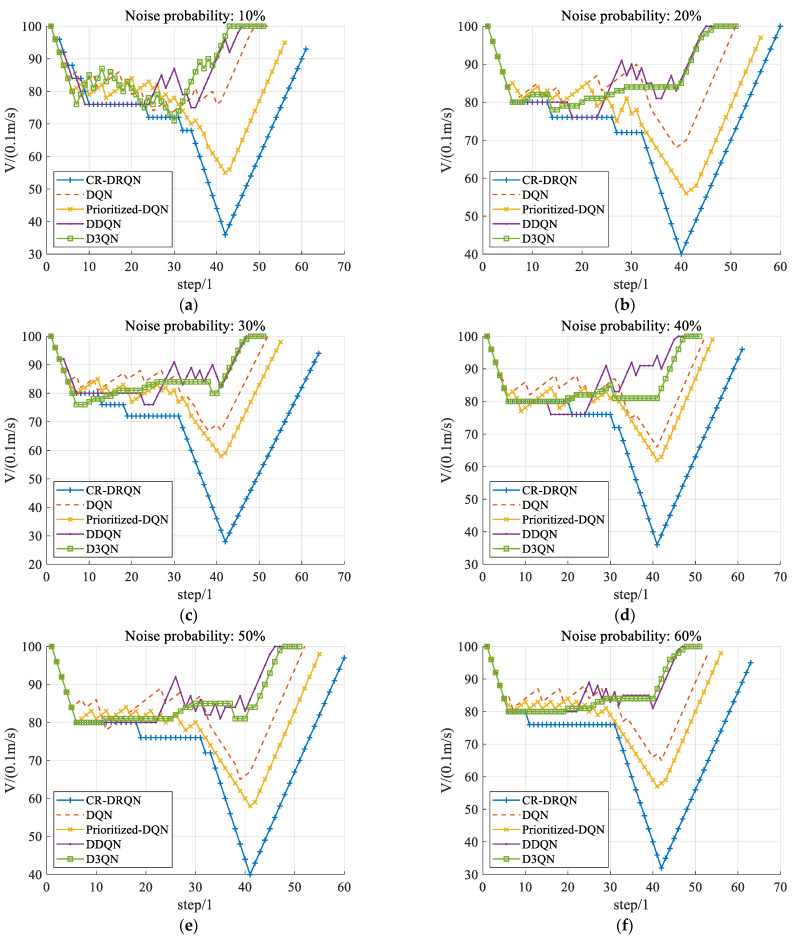
Velocity of an ego vehicle through an intersection at different noise probabilities. (**a**–**f**) present the results at the noise probabilities of 10–60% respectively.

The ability to predict collision risk has a significant impact on decision success. This ability is reflected by the velocity change of the ego vehicle. The velocity of the ego vehicle is recorded from the above safety evaluation experiments. In those experiments, decisions of DQN, Prioritized-DQN, DDQN, D3QN, and CR-DRQN are successful. As shown in Figure 6, with different probabilities of noise occurrence, the velocity of the ego vehicle slows down before an intersection because it is affected by the safety reward *R_safe_*. When surrounding vehicles leave the intersection, the ego vehicle accelerates to maximum velocity under the influence of *R_efficient_* until the episode ends. In Figure 6, although DDQN is aware of dangers ahead, deceleration action is brief. Then DDQN speeds up soon. This demonstrates that DDQN has a poor ability to avoid a secondary collision. Moreover, the result of D3QN is like DDQN. The intelligent land vehicle decelerates to about 8 m/s first but the deceleration is brief. This illustrates that D3QN has a poor capability to avoid second collisions, too. In addition, in the first 4 s, the velocity of the ego vehicle based on CR-DRQN drops to approximately 4 m/s while the velocities of DQN and Prioritized-DQN are approximately 7 m/s and 6 m/s, respectively. This shows that the deceleration of DQN and Prioritized-DQN are less than CR-DRQN in the first four seconds. Thus, the ego vehicle keeps a higher velocity based on DQN and Prioritized-DQN before the intersection. However, it is too difficult to avoid a collision with high velocity. To conclude, both DQN and Prioritized-DQN have weak abilities of collision risk prediction. On the contrary, an intelligent land vehicle based on CR-DRQN can drive through an intersection safely with lower velocity before an intersection. This verifies that CR-DRQN’s ability to detect collision danger is more outstanding than other algorithms. That is also why CR-DRQN’s decision-making success rate is higher.

When the environment is partially observable on account of sensor noise, the observed states exhibit hysteresis and are different from the environmental states. In this case, there is no collision risk warning from the state input, but collision risk exists. Because of weak abilities to predict risks, DQN, Prioritized-DQN, D3QN, and DDQN are highly dependent on the accuracy of environmental perception. Therefore, the success rates of decisions are greatly affected by perception error owing to the sensors. Nevertheless, CR-DRQN trains time series so that the ability of risk prediction is stronger than the other four algorithms. Although the environment is perceived with different levels of noise, the success rate of CR-DRQN decision-making is slightly affected. The decision-making performance of CR-DRQN in a partially observable environment is superior to DQN and its variants. That is, the probability of decision failure is greatly reduced in the condition of sensor noise.

#### 5.3.3. Comfort Evaluation 

**Figure 7:**
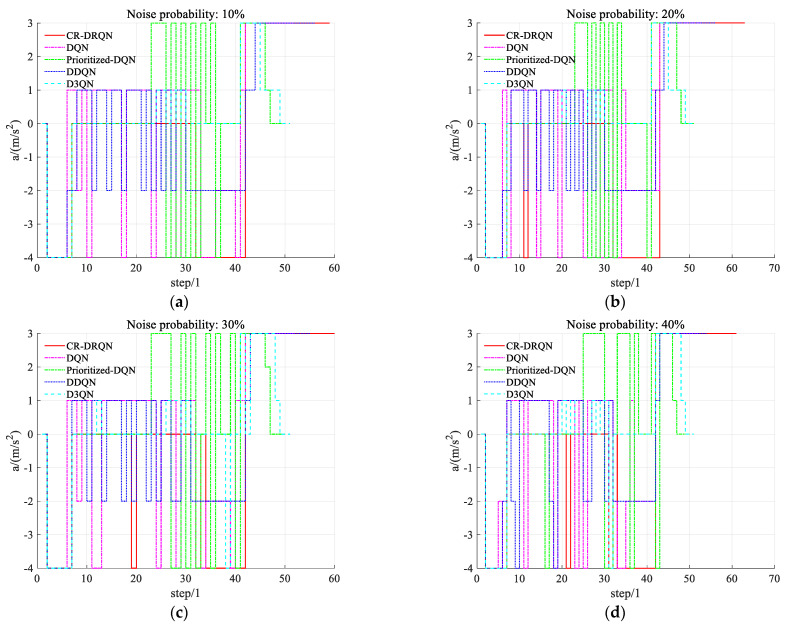

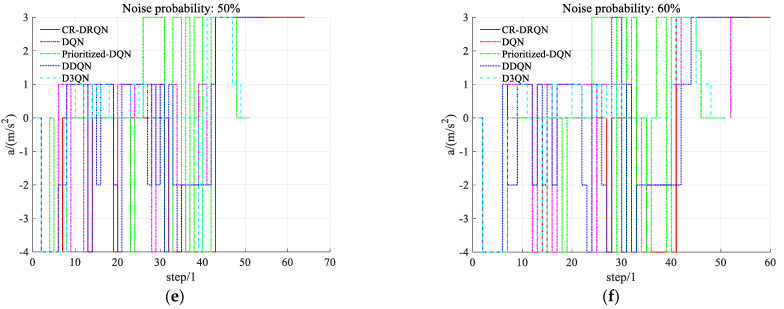
Action chose by CR-DRQN at different probabilities of noise occurrence. (**a**–**f**) present the results at the noise probabilities of 10–60% respectively.

Here, the comfort of decision-making is tested by the frequency of acceleration change. The acceleration of an intelligent land vehicle is recorded from the above safety evaluation experiment with successful decisions. Acceleration curves with decision steps under different probabilities of noise occurrence are shown in Figure 7. This shows apparently that the acceleration fluctuation of CR-DRQN is smoother than other algorithms at different probabilities of noise occurrence. The velocity control of CR-DRQN is more stable; thus, driving comfort is enhanced.

To intuitively show the frequency of acceleration change, Table 3 records the average change times of acceleration based on CR-DRQN and other algorithms at different probabilities of noise occurrence under 30 experiments. The acceleration change of CR-DRQN is less than 16 times, while others are more than 22 times, even up to 44 times. With the increase in probability of noise occurrence, CR-DRQN has a great ability to maintain a low frequency of acceleration change. However, DQN and its variants keep a high frequency of acceleration change. Because of sensor noise, the observed states are not accurate. In this case, it is hard for an intelligent land vehicle to make the right predictions. The ego vehicle may predict collision risk sometimes or detect danger that passes soon. This leads to a high frequency of acceleration change. Nevertheless, CR-DRQN can keep the low frequency of acceleration change because it can train the time series by LSTM. CR-DRQN guarantees the safety and comfort of decision-making for intelligent land vehicles.

To sum up, the above experiments verify the effectiveness of CR-DRQN in driving behavior decision-making of intelligent land vehicles at the disorderly intersection. CR-DRQN successfully predicts collision risk, makes driving behavior decisions on time, and passes an intersection quickly after collision risk is eliminated. At the same time, when there is sensor noise in the environmental state input, decision performance is still high.

## 6. Conclusions

At a real disorderly intersection, the observed states are different from the environmental states owing to sensor noise. This easily causes a partially observable environment and decision failure. In this study, a collision relationship-based driving behavior decision-making method for an intelligent land vehicle via DRQN (CR-DRQN) is proposed. The input of CR-DRQN is defined as the collision relationship between intelligent land vehicles and other vehicles to enhance the generalization of the input state. Then, CR-DRQN uses LSTM to replace the first full connection layer of DQN, with the ability of training time series to improve the danger prediction ability under sensor noise. Finally, a series of experiments verify that CR-DRQN shows better performance than traditional DQN and its variants, in the aspect of safety, ability of risk prediction, and comfort.

In future work, we will have an intelligent land vehicle learn to make complex decision-making decisions with expected trajectories based on reverse reinforcement learning.

## Figures and Tables

**Table 1:** Summary of the common decision-making method.

Method		Reference	Application
**Game theory-based**		[7]	Lane changing at congested, urban scenarios
		[8]	Decision-making at an urban unsignalized intersection
**Generative** **decision-making**		[9]	Parking
**Fuzzy decision-making**		[10]	Decision-making in a vehicle sensor tracking system
		[11]	Lane changing
**Partially observable** **Markov decision-making**	Machine learning	[12]	Driving style classification
		[13]	Lateral motion prediction
	Deep reinforcement learning	[14]	Decision-making at intersections
		[15]	Lane changing in dynamic and uncertain highways

**Table 2:** Settings of CR-DRQN’s parameters and corresponding training results.

SerialNumber	Network Layers	Network Parameters	*R_safe_*	*R_comfort_*	*R_efficient_*	Q-Value
1	4	64/32/16/5	−5	−0.2	442.7	83.34
2		128/64/32/5	9	−1.6	466.4	100.68
3		256/128/64/5	12	−1.4	447.8	100.26
4	5	64/32/16/8/5	3	−0.4	448.3	92.26
5		128/64/32/16/5	−4	−0.6	446.6	84.72
6		256/128/64/32/5	3	−6.4	444.8	85.56
7	**6**	128/64/32/16/8/5	3	−0.4	448.3	92.26
8		160/80/40/20/10/5	3	−0.4	448.3	92.26
**9**		**256/128/64/32/16/5**	**30**	**−0.8**	485.5	**126.3**
10		320/160/80/40/20/5	30	−1	485.5	126.1
11	7	256/128/64/32/16/8/5	−15	−11.6	439	61.2
12		320/160/80/40/20/10/5	15	−4.3	**554.9**	121.68
13		512/256/128/64/32/16/5	26	−0.5	474.7	120.44
14		640/320/160/80/40/20/5	6	−0.4	443.4	94.26
15	8	512/256/128/64/32/16/8/5	−3	−8.2	437	76.2
16		640/320/160/80/40/20/10/5	−9	−0.7	434.4	77.18

**Table 3:** Average change times of the ego vehicle’s acceleration at different probabilities of noise occurrence after 30 experiments.

Noise Probability	10%	20%	30%	40%	50%	60%
DQN	33	36	28	36	40	25
Prioritized-DQN	40	37	40	33	41	44
DDQN	38	32	35	32	32	38
D3QN	22	23	32	29	32	40
**CR-DRQN**	**9**	**12**	**15**	**16**	**16**	**16**
